# Response of Arugula to Integrated Use of Biological, Inorganic, and Organic Fertilization

**DOI:** 10.3390/microorganisms12071334

**Published:** 2024-06-29

**Authors:** Aleksandra Stanojković-Sebić, Vladimir Miladinović, Olivera Stajković-Srbinović, Radmila Pivić

**Affiliations:** 1 Institute of Soil Science, Teodora Drajzera 7, 11000 Belgrade, Serbia; soils.stanojkovicsebic@gmail.com (A.S.-S.); oliverastajkovic@yahoo.com (O.S.-S.); 2 Institute for Vegetable Crops, Karadjordjeva 71, 11420 Smederevska Palanka, Serbia; vladimir.miladinovic33@gmail.com

**Keywords:** arugula, different fertilizations, bacterial strains, Vertisol, plant nutrients, yield

## Abstract

This study evaluated the effects of solely and integrated application of inorganic (NPK), commercial organic (NC), and biological (MIX, mixed strains *Ensifer meliloti* and *Azotobacter chroococcum*) fertilizers on the chemical characteristics of arugula biomass and its yield, as well as changes in soil microbiological parameters after the experiment in relation to the control treatment (Ø). The experiment was performed in semi-controlled greenhouse conditions, in pots, from the 4th decade of March to the 2nd decade of September, in 2023, at three cutting times/swaths, during one agricultural season, with Vertisol soil. For soil characterization, the following parameters were analysed: granulometric composition using sieving and sedimentation procedure; soil acidity—potentiometrically; SOM—soil organic matter by Kotzmann method; total N using CNS analyser; available P—spectrophotometrically; available K—flame photometrically; total number of microorganisms on an agarized soil extract medium; fungi on a solid Czapek agar; actinomycetes on a solid Krasiljnikov agar with saccharose; *Azotobacter* spp. on a liquid Fyodorov medium with mannitol; ammonifiers on a liquid medium with asparagine; and dehydrogenase activity—spectrophotometrically. For plant characterization, the following parameters were determined: N and C, both on CNS analyser; P on spectrophotometer; K on flame photometer; air-dried yield biomass. A stimulative effect on all microbiological parameters was found in the treatment with integrated use of organic and biological fertilizer, except for fungi, which grew better in the treatments with separate inorganic and organic fertilizers. Generally, the stimulative impact on plant chemical parameters manifested in combined inorganic and biological, organic and biological, and inorganic and organic fertilization treatments, and was inhibited in treatment without fertilization, in all three swaths, which could also be stated for the plant yield. Positive influence of all fertilization treatments on chemical parameters was observed for the second swath in relation to the first and the third. The total yield in the NPK+MIX treatment was 121%, and in the NC+MIX treatment, it was 87% higher compared to the control (Ø). In general, integrated use of inorganic and biological, organic and biological, and inorganic and organic fertilizers, respectively, could be proposed as an optimal fertilization treatment in arugula cultivation.

## 1. Introduction

Arugula (*Eruca sativa* Mill.) is an annual or biennial vegetable herb that belongs to the cabbage family (Brassicaceae). It is very medicinal, as it is rich in vitamins and inorganics, benefiting the health. Arugula leaves contain a high level of folic acid and antioxidants, such as vitamins A, C, and K, so arugula is also known as a very successful “fighter” against free radicals. These vitamins have a beneficial effect on improving the condition of the bones, teeth, eyes, and lungs. The plant is full of fibre, which supports good digestion, causing a feeling of satiety. It regulates the sugar level in the blood and is rich in carotenoids and many minerals, such as potassium, manganese, iron, and calcium, all of which are very useful in human nutrition [1]. Due to its antioxidant activity and the abundance of antioxidants, this vegetable helps strengthen brain functions, improves metabolism, and strengthens the immune system by suppressing pathogenic microorganisms. As a medicine, arugula is considered a natural antibiotic, is used to treat ulcers, osteoporosis, chronic constipation, colds and coughs, has antidiabetic and antihypertensive activity, and regulates digestion [2,3]. Arugula is also an aromatic plant which, due to its bitter flavour, gives salads a special taste. In the cultivation of arugula, the rule for the plant is that the more its leaves are cut, the faster its growth. The plant has modest heat requirements, is resistant to frost (up to −4 °C), tolerates drought very well, likes partial shade from the sun, and usually does not need special care, which is why it can be grown all year round. It thrives the best on light sandy, medium heavy, wet, and dry soils with a neutral pH, although it can also be successfully grown on soils with an alkaline pH. At the same time, it is more sensitive to acidic soils. Arugula can be grown in open fields, but one of the simplest ways to grow arugula is by planting it in pots. In arugula growing, fertilization is the second most important cultivation requirement after irrigation [4]. 

Vertisol is one of the most widespread types of soil in Serbia [5]. Numerous authors who have studied this type of soil [6,7] have pointed out its limitations from the perspective of an unfavourable water–air and thermal regime, heavy mechanical composition, and unfavourable structure, both in terms of the shape and size of aggregates and their stability. Its chemical properties are far more favourable. It is characterized by a large adsorption capacity, where the alkaline saturation at the base is up to 90%, and a generally neutral to weakly alkaline pH, medium to well supplied with soil organic matter, total nitrogen, and available potassium, and quite low provided with available phosphorus. Therefore, more emphasis is placed on the application of fertilizers with a higher proportion of phosphorus.

Inorganic fertilizers are considered to be a powerful “lever” in intensifying agricultural production only if they are applied rationally. Intensification of agricultural production increases the amount of inorganic fertilizer used per unit area. This increases the efforts for their more rational use to manifest their positive functions to the maximum [8]. The wide application of inorganic fertilizers in agriculture is conditioned by the low supply of plant nutrition elements, especially nitrogen. Inorganic and organic fertilizer reduction has a significant positive consequence in reducing carbon emissions and environmental pollution [9]. Some of the common ways to reduce the use of inorganic fertilizers are the promotion of soil fertility testing techniques, the replacement or partial replacement of inorganic fertilizers with organic or biological fertilizers, or the improvement of soil fertility using a variety of fertilizer mixtures with different fertilization regimes [10]. Appropriate application of organic and biological fertilizers simultaneously with inorganic fertilizers can improve soil properties and transform bacterial and fungal ecology, thus improving the yield and quality of cultivated crops [11]. 

The use of organic fertilizers from different materials such as farmyard manure, sewage sludge, biogas digestates, compost, urban compost, animal manure (from cattle, pigs, poultry, sheep, goats, and other livestock) and other components that contain certain inorganic nutrients for crop plants can increase soil water-holding capacity and improve soil texture by delivering organic matter to the soil. Organic fertilizers also increase the contents of some heavy metals such as Zn and Fe in vegetables [12]; the highest contaminations with Pb, Zn, Ni, Cr, and Hg have been found in sewage sludge, particularly in industrial sludge. High concentrations of Cd, Cu, Zn, and As is Arsenic can be found in pig manure [13]. Changes in organic management, such as the use of commercial organic fertilizers of animal origin, could have a high potential in crop production. By substituting inorganic for organic fertilizer, such as poultry faeces or cow dung, the soil microbial community composition is changed, which contributes to soil ecology optimization [14]. According to Abou-Sreea [15], organic fertilizers can entirely or partially replace chemical fertilizers. The previous results [16,17,18] indicated that partial substitution of inorganic for organic fertilizer positively affected maize grain and rice yield, reducing the need for chemical nitrogen fertilizers. The use of biological fertilizers that include effective microorganisms and eco-friendly substances could minimize the application of inorganic fertilizers, maintain and improve soil properties and fertility, and increase crop productivity. These fertilizers contain plant growth-promoting microbes such as nitrogen-fixing bacteria, used as soil or seed inoculants, and can stimulate the availability of the main plant nutrients [19,20]. Compared to inorganic and organic fertilizers, the use of biological fertilizers can increase the levels of organic carbon, improve soil microorganisms’ diversity, increase the availability of plant nutrients, and have a significant impact on crop yields [21]. Integrated use of biological and organic fertilizers indicated positive results, where the compost would act as a long-term reserve and source of macronutrients [22] in a study related to four cutting processes in one agricultural season [23]. Previous research [24] has indicated effectivity in the integrated use of chemical, organic, and biological fertilizers in vegetable production, meaning that biological fertilizers are not a complete substitute for chemical ones.

In the conditions of Serbia, the rationalization of inorganic fertilizer use has a pronounced social significance due to the import of increasingly expensive and deficient raw materials for their production. In addition, inorganic fertilizers show differing effects in different soil–climatic conditions and at different agrotechnical levels of plant production [8]. To analyse soil microbiological activity, chemical parameters, and plant yield in the experimental vegetative cultivation of arugula under different fertilization treatments when using soil with a heavy mechanical composition and a high clay fraction, the Vertisol type of soil was selected as the soil material in the present study. 

It is a fact that intensive agricultural production is based on the use of different types and amounts of inorganic and organic fertilizers. Knowing the influence of these fertilizers on soil microorganisms, it can be expected that they will have a different effect on microbiological activity during the growing season. Since vegetable crops, including arugula, occupy an important place in vegetable production, this study has the task of determining the types of rational fertilization with inorganic and organic fertilizers, with the possibility of applying microbiological inoculants (biological fertilizers). The conducted research allows us to propose an optimal fertilization variant, the application of which will not seriously disturb the biological balance in the soil, through which it would be possible to maintain or increase the productivity of the soil, the quality of the yield, and the production of healthy food. 

The main objectives of this study were to examine the effects of sole and integrated use of inorganic [composite NPK (15:15:15)], commercial organic (solid NPK 4:3:4 nutrient of animal origin—Nervosol Complex), and biological (mixed bacterial strains *Ensifer meliloti* and *Azotobacter chroococcum*) fertilizers on the following parameters: the significance of soil microbiological parameters changes after the end of the experiment in relation to non-fertilized soil; main chemical characteristics of aerial biomass of arugula (*Eruca sativa* Mill.), its total yield, yields in three swaths, during one agricultural season.

## 2. Materials and Methods 

### 2.1. Experimental Design 

The research was performed in greenhouse vegetative conditions using plastic pots, from the 4th decade of March to the 2nd decade of September, in 2023. Each pot was filled with 1.4 kg pot^−1^ of homogenized Vertisol soil [25], brought from the field in the Mala Ivanča settlement, Sopot Municipality (grid reference: 44°35′ N, 20°36′ E), located about 35 km from Belgrade in Serbia. In every plastic pot, ten arugula seeds were sown on 30 March. Arugula plants were grown according to the standard growing methods (watering and regular manual weed control) and without the application of any plant protection products. The study with the same plant was repeated two more times, without re-sowing or re-treatment, and with three cuts of plants during one agricultural season, as follows: swath I—13 June 2023; swath II—25 July 2023; swath III—14 September 2023. The designed treatments, set up in three replications, and their abbreviations, are given in Table 1.

The rates of applied doses of inorganic fertilizers are common in vegetable production in the field of study (60 kg N ha^−1^, 60 kg P_2_O_5_ ha^−1^, 40 kg K_2_O ha^−1^). Inorganic [composite NPK (15:15:15)] fertilizer was applied as follows: nitrogen (N) fertilizer, in the form of urea with 46% N; phosphorus (P) fertilizer in the form of monoammonium phosphate with 22.16% P and 11% N (52% phosphorus in the form of P_2_O_5_); potassium (K) fertilizer in the form of potassium chloride with 33.20% K (40% in the form of K_2_O). Organic fertilizer was used as a solid NPK 4:3:4 nutrient of animal origin, commercially called Nervosol Complex, with the following main chemical composition: 4% total N, 4% organic N, 1.28% P (3% P in the form of P_2_O_5_), 3.32% K (4% in the form of K_2_O), and 30% organic carbon (C) [26]. Before sowing, the amount of inorganic and organic fertilizers was measured according to the experimental design and mixed with soil (recalculated as for 1 kg of soil): NPK inorganic fertilizer (15:15:15) = 0.17 g kg^−1^; NC organic fertilizer = 0.06 g kg^−1^. After sowing, the application of biological fertilizers involved the inoculation of soil and arugula seeds in pots with a mixed culture of bacteria *Ensifer meliloti*, strain 218, and *Azotobacter chroococoum*, strain A1. Both strains are from the Microbiology Department of the Institute of Soil Science in Belgrade. The bacterial strain *Ensifer meliloti* 218 was grown in yeast mannitol broth (YMB) according to the method of Vincent, as cited by Shimoia et al. [27]. *Azotobacter chroococoum* A1 was grown in a Fyodorov liquid medium according to the method of Govedarica and Jarak, as cited by Stajković-Srbinović et al. [28]. Both strains were incubated at 28 °C in a thermostat for 48 h on an orbital shaker at 150 rpm and used for soil and seed inoculation in a mixture, in the amount of 150 mL for each strain. Concentrations of used cultures were 10^9^ CFU mL^−1^, determined by plating of decimal culture dilutions on YMB and Fjodorov medium. The inoculation of the soil and arugula seeds in each pot was conducted with 10 mL of liquid-mixed bacterial inoculum, and the control was treated with distilled water in the same amount as the treatments where the bacterial mix was used. This type of soil and seed treatment in pots were carried out according to our previous research [29]. 

### 2.2. Soil Preparation and Analysis

The preparation of soil samples for physico-chemical analysis was performed using the standard method [30], where the samples were air-dried, crushed, and passed through a sieve (≤2 mm). Sampling, handling, and storage of soil for microbiological analysis were carried out using standard methods [31]. Accordingly, the average soil sample for the initial (non-vegetation) phase was taken from the field aseptically with a probe several times from a depth of 0–20 cm, in the amount of 1.0 kg, and transferred in loosely tied polyethylene bags. The soil was transported within a short time to the laboratory in a manner that minimized all possible changes. The soil was analysed immediately after being brought from the field, whereupon vegetation, larger soil fauna, and stones were removed the soil was passed through a 2 mm sieve, thus maintaining the aerobic nature of the soil. 

An observation of the soil material included determination of soil granulometric and chemical composition (both before setting up the experiment), and microbiological parameters (before and after the experiment). Soil granulometric composition (the share of clay, sand, and silt fractions) was performed by determination of particle size distribution in mineral soil material, using sieving and sedimentation [32]. Accordingly, using the texture triangle of the International Union of Soil Science (IUSS) the textural soil class was found [33]. Chemical soil properties were determined using the following chemical analyses: soil acidity (active: pH in H_2_O and substitutional: 1M KCl; *v*/*v*: soil: H_2_O = 1:5, soil:1M KCl = 1:5) was analysed potentiometrically using a glass electrode [34]; the content of soil organic matter (SOM) was determined using the humus method of Kotzmann, according to Jakovljević et al., as cited by Milivojević et al. [35]; total N was analysed by dry combustion using elemental a Vario EL III CNS analyser (Elementar Analysensysteme GmbH, Langenselbold, Germany), according to the standard method [36]; available P and K were analysed according to the AL method of Egner-Riehm, as cited by Latković et al. [37], where P was determined spectrophotometrically after colour development with ammonium molybdate and stannous chloride, and K photometrically by flame. The number of microorganisms was determined according to the dilution method on the appropriate nutritive medium by using the decimal dilutions (10^−1^–10^−8^), after the method of Pochon and Tardieux, as cited by Laid et al. [38]. The values were expressed per g of absolutely dry soil, using the calculation of the moisture content in the soil sample, gravimetrically, according to the principle of heating or drying the samples to evaporate water from them [39]. For the assessment of microbiological activity in the soil [40], the total numbers of microorganisms, fungi, actinomycetes, *Azotobacter* spp., and ammonifiers were determined as the basic soil microbiological criteria. The total number of microorganisms was analysed using an agarized soil extract medium, sowing solid nutrient media with decimal dilutions of the tested soil suspension. Grown colonies on the medium were counted after seven days of incubation in a thermostat at 28 °C ± 1. The number of soil fungi was determined by the indirect method of agar plates on a Czapek agar, based on the principle of sowing a solid nutrient medium with decimal dilutions of the tested soil suspension. The sown medium was incubated for five days in a thermostat at 28 °C ± 1, after which the grown colonies were counted. The number of actinomycetes was determined by an indirect method of agar plates on a substrate with sucrose according to Krasiljnikov, sowing solid nutrient media with decimal dilutions of the tested soil suspension, its incubation for seven days in a thermostat at 28 °C ± 1, and the counting of grown colonies. The number of *Azotobacter* spp. was determined using a liquid medium with mannitol, per Fyodorov. After seven days of incubation in a thermostat at 28 °C ± 1, the results were read by determining the number of positive test tubes in which a cover, scrum, or membrane appeared on the surface of the medium; these were initially colourless, then yellowish, and later dark. Based on the number of positive tubes in each dilution, the number of *Azotobacter* spp. was calculated using the McCrady table. The number of ammonifiers was determined by the indirect method in a liquid medium with asparagine as the source of nitrogen, by sowing liquid nutrient medium with decimal dilutions of the tested soil suspension. After incubation in a thermostat at 28 °C ± 1 for seven days, the increase in the number of bacteria was examined by visual evaluation of the medium turbidity. The results were read using Nessler’s reagent, and test tubes in which a positive reaction to ammonia was observed in the form of an orange-coloured solution were noted. Based on the number of positive tubes in each dilution, the number of ammonifiers was calculated using the McCrady table. Enzymatic activity was evaluated using soil dehydrogenase activity (DHA). This was determined spectrophotometrically under standard conditions, which implied 24 h of incubation at 30 °C ± 1, at a wavelength of 546 nm, by measuring the intensity of the red-coloured triphenyl formazan (TPF) extinction, which was formed by reduction of 2,3,5-triphenyl tetrazolium chloride. The standard curve with concentrations of 0.25, 0.5, 1.0, 1.5, and 2.0 mg of TPF, was used for the TPF quantity reading. The obtained values of DHA were expressed in µg TPF g^−1^ of an air-dried soil [41].

### 2.3. Plant Preparation and Analysis

The aerial biomass (stems with leaves) of arugula plants was taken at the beginning of rosette formation from each experimental variant, replicated, and, at three cutting times during one agricultural season, air-dried and measured for yield (g pot^−1^). It was additionally dried for 2 h at 105 °C ± 1 and weighed again for chemical analysis, using the gravimetric method for the determination of the dry matter content of plant tissue [42]. An elemental CNS analyser, Vario model EL III, was used in the determination of N and C content [36]. Contents of P and K were determined using “wet” combustion, where the plant biomass was heated to boiling with a mixture of concentrated H_2_SO_4_ and HClO_4_, where P was determined spectrophotometrically with molybdate, and K photometrically by flame [43,44]. 

### 2.4. Statistical Analysis and Graphical Presentation

The obtained data on the granulometric composition and the chemical and microbiological parameters of the soil before setting up the experiment are presented as arithmetic means of three replicates and standard deviation values; the effect of treatments on microbiological parameters was statistically processed at the end of the experiment. Statistical processing of the treatment effects on plant yield and chemical parameters at all three cutting times were evaluated using analysis of variance (SPSS 23.0, Chicago, IL, USA), followed by Duncan’s multiple range test (DMRT). A graphical presentation of the obtained results was provided using Excel.

## 3. Results

### 3.1. Soil Physical, Chemical, and Microbiological Properties 

The studied soil material of Vertisol (initial, non-vegetation phase) is characterized by acid reaction, high available K, very low available P, medium total N, and SOM supply, with relatively good microbiological activity. According to granulometric composition, Vertisol is a light clay with an increased share of the clay fraction and has a relatively favourable particle size distribution for the cultivation of arugula (Table 2).

### 3.2. Effect of Fertilization on Soil Microbiological Parameters 

Experimental data on the studied fertilization treatment effects on the mean values of the microbiological parameters in soil under arugula are given in Table 3. Different fertilization treatments were the main factor in the statistical data analysis.

The obtained results of the analysed microbiological parameters in tested soil indicate a highly statistically significant (*p* < 0.01) increase in the total number of microorganisms, actinomycetes, and *Azotobacter* spp. and dehydrogenase activity (DHA) in the variant, which included an integrated use of organic NC fertilizer and biological mixture (variant NC+MIX) compared to the other tested treatments, especially the treatment without fertilization. A statistically significant (*p* < 0.05) increase in the number of fungi was found in the treatments NPK and NC, particularly compared to the non-fertilized and MIX variants. A noticeable but statistically insignificant (*p* > 0.05) increase in the number of ammonifiers relative to the other variants was found in the treatments which included a combination of an inorganic NPK and organic fertilizers and biological mixture (variants NPK+MIX and NC+MIX). 

### 3.3. Effect of Fertilization on Chemical Composition of Arugula

By analysing the chemical parameters, including N, P, K, and C, in aerial biomass of arugula plants during one agricultural season in three cutting times (swath I, swath II, swath III), it was generally determined the significance in differences in these parameters depending on applied fertilization treatments (Table 4, Table 5 and Table 6). Different fertilization treatments were the main factor in the statistical data analysis. 

#### 3.3.1. The First Cutting Time—Swath I

The obtained results of the analysed chemical composition in tested plant biomass of the first swath are presented in Table 4. 

The results indicate a statistical significance in the fertilization effect of different treatments on tested parameters, except for the content of C, in which an insignificantly (*p* > 0.05) slight increase in NPK+MIX treatment was found. A statistically significant (*p* < 0.05) increase in the content of N was found in the NPK+MIX treatment, while a noticeable decrease was observed for the treatment that included NPK, compared to the other tested treatments. Concerning the others, a highly statistically significant (*p* < 0.01) increase in the content of P was recorded in variants with integrated use of inorganic NPK and organic fertilizer (NPK+NC); in variants with integrated use of biological mixture and organic NC fertilizer (NC+MIX) and of biological mixture and inorganic NPK fertilizer (NPK+MIX), it was observed a highly significant (*p* < 0.01) increase in the K content.

#### 3.3.2. The Second Cutting Time—Swath II

The obtained data on the tested chemical composition in the investigated plant biomass of the second swath are presented in Table 5.

Accordingly, the existence of statistical significance in the fertilization effect of all treatments on tested plant chemical parameters was observed. The content of N and K was highly significantly (*p* < 0.01) increased in the NPK+MIX and NC+MIX treatments compared to the others, while the content of N was highly significantly (*p* < 0.01), decreased in the NPK+NC treatment in relation to the other tested treatments. A statistically significant (*p* < 0.05) increase in the content of C was observed for the NPK+MIX and non-fertilized treatment. A highly significant (*p* < 0.01) increase in P content was recorded in NPK+NC treatment in relation to the others; its highly significant (*p* < 0.01) decrease was found from the treatment without fertilization.

#### 3.3.3. The Third Cutting Time—Swath III

The obtained results of the analysed chemical parameters in tested plant biomass of the third swath are presented in Table 6.

There were significant differences among the fertilization treatments for all arugula chemical parameters. The highest and statistically highly significant (*p* < 0.01) stimulating effect of the NPK+MIX treatment was observed for the contents of N, C, and K, and NPK treatment for K, NC+MIX treatment for P, and non-fertilized treatment for C content, respectively. A highly statistically significant (*p* < 0.01) decrease in the content of N and P was found in the non-fertilized treatment and in the NC and MIX treatments, respectively, for K, in relation to the other tested variants. 

### 3.4. Effect of Cutting Time—Swath and Different Fertilizers on Arugula Chemical Composition 

**Figure 1:**
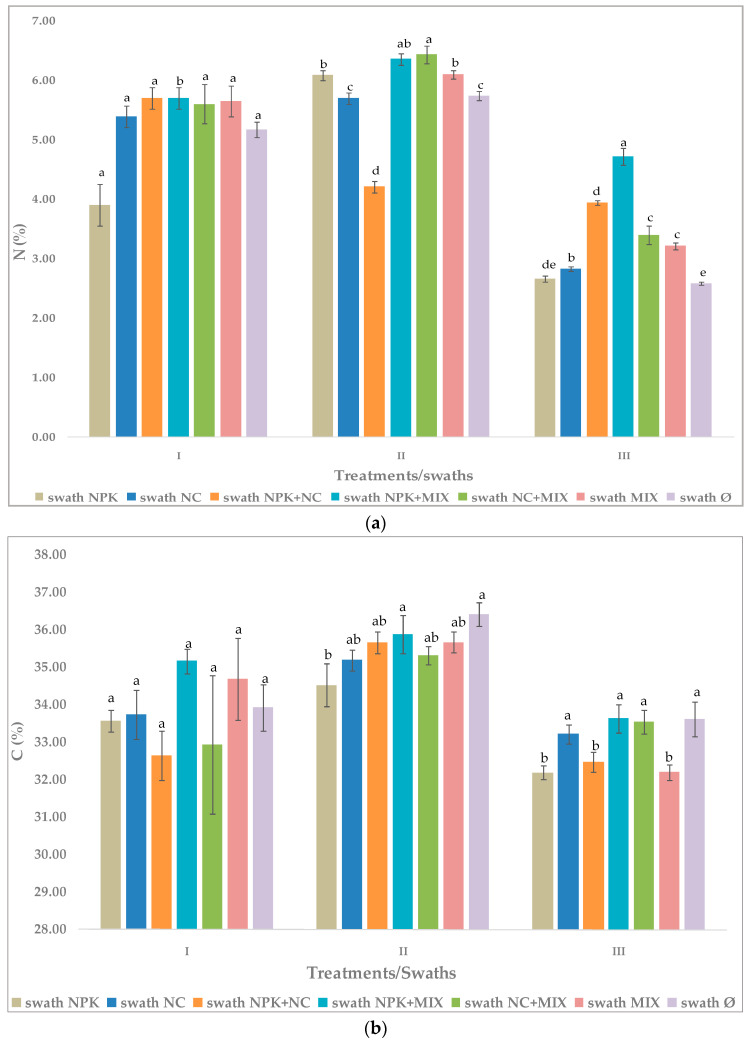

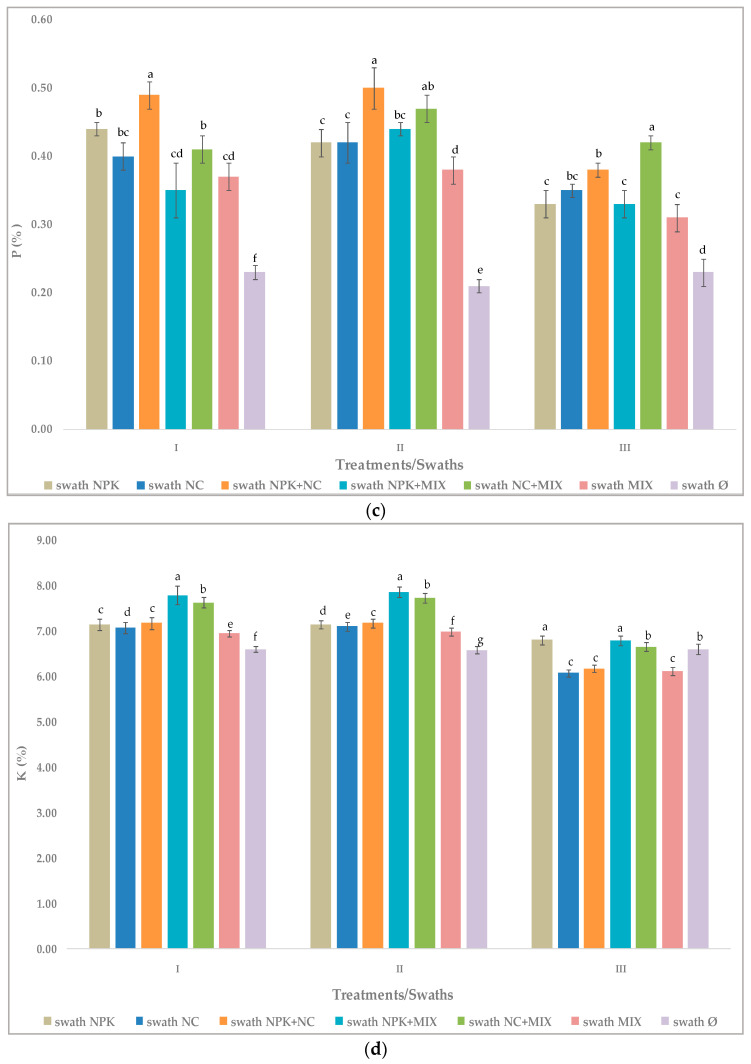
Effect of fertilization treatments on the average values of the arugula chemical parameters, depending on the time of cutting—swath: (**a**) Effect on N. (**b**) Effect on C. (**c**) Effect on P. (**d**) Effect on K. fertilization treatments and their abbreviation names are given in Table 1; the values shown in the histogram represent the average ± standard deviation; DMRT at *p* ≤ 0.05—values followed by the same letter in the histogram bar are not significantly different.

Experimental data on the studied effects of fertilization treatments on the average values of the arugula chemical parameters, depending on the time of cutting—swath, during one agricultural season, indicate the differences in these parameters (Figure 1). 

A very noticeable increase in the amount of N was observed in the second swath compared to the first and especially the third regarding the treatment with inorganic NPK. A stimulative effect of the NC, MIX, NPK+MIX, and no fertilization treatments on N was noticed in the second and first swath in relation to the third. Conversely, a lower content of N was observed in the second swath for the NPK+NC treatment compared to the first, but higher than the third. The lowest concentrations of N were found in the third swath for all the tested treatments. Slightly higher C values were found in the second swath in all the tested treatments compared to the first and the third swath. A slightly stimulative effect of NPK+MIX and NC+MIX on P and K concentrations was observed in the second swath in relation to the first and the third, whereby the values of these parameters were almost uniform in the first and the third swath. Decreased content of P and K was observed in the no-fertilization treatments for the first and the second swath.

### 3.5. Effect of Fertilization Treatments on Yield of Arugula 

By analysing the yield of aerial biomass of arugula plants during one agricultural season in three cutting times (swath I, swath II, swath III) and the total yield, it was determined that the differences in this parameter depend on applied fertilization treatment (Table 7). Different fertilization treatments were the main factor in the statistical data analysis.

There were highly significant differences (*p* < 0.01) in the yield of arugula among the fertilization treatments, which included integrated use of biological mixture and inorganic NPK fertilizer (NPK+MIX) in relation to other treatments for the first swath. A noticeable but insignificant stimulative effect on yield was observed in the treatment with organic fertilizer and biological mixture (NC+MIX). In addition, a low yield was observed in the treatment with bacterial mixture, and the lowest in non-fertilized treatment.

For the second swath, a statistically insignificant (*p* > 0.05) fertilization effect on the yield of arugula was recorded in all treatments. A slightly stimulative but insignificant effect on yield was found in the NPK+MIX and NC+MIX treatments, while the lowest and insignificant (*p* > 0.05) yield was observed in the treatment without fertilization (control), and a low effect in the MIX treatment. 

It was observed a noticeable but insignificant effect (*p* > 0.05) of all fertilization treatments on arugula yield, for the third swath. In the NPK+MIX, NC+MIX, NPK, and NC treatments, a slightly stimulative but insignificant effect was found on the yield. A low and the lowest yield were observed in the treatment with no fertilization and the treatment with bacterial mixture, respectively, with the lack of statistical significance (*p* > 0.05).

Total arugula yield (swath I + swath II + swath III) was significantly (*p* < 0.05) higher in relation to other treatments in the fertilization treatment which included integrated use of biological mixture and inorganic NPK fertilizer (NPK+MIX). which was also observed for noticeable but insignificant effect (*p* > 0.05) of the treatment with organic fertilizer and biological mixture (NC+MIX). The lowest total yield was observed in the treatment without fertilization. 

**Figure 2:**
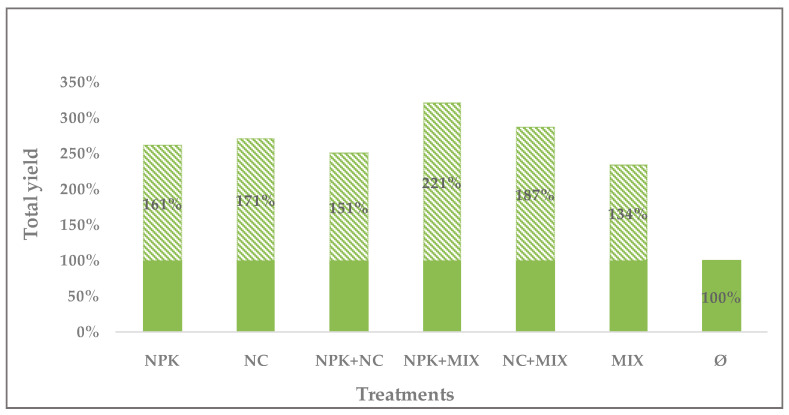
Percentage increase in the total yield per treatment compared to the non-fertilized variant.

In relation to the non-fertilized variant (Ø), percentage increase in total yield per treatment was as follows: 121% for NPK+MIX, 87% for NC+MIX, 71% for NC, 61% for NPK, 51% for NPK+NC, and 34% for MIX (Figure 2).

## 4. Discussion

Fertilization in modern agricultural production aims not only to maintain but also to improve the quantity and quality of the cultivated plants’ yield. Research in this area is most often directed towards increasing the yield of agricultural crops, while their negative effect on changes in soil and plant chemical and soil biological properties is often neglected. In addition to higher plant productivity, intervention with various organic and biological fertilizers achieves the sustainability of agricultural production from the aspect of increasing the availability of nutrients for plants, their nutritional value, and yield [45,46]. 

Vertisol soil, used in the present study, was determined to be a clay loam, but with heavy mechanical composition, having an increased clay fraction, with favourable chemical properties (except for the acid pH and quite low content of available P) and microbiological activity. Similarly, by examining the loam type of soil originating from the Šumadija district, Kragujevac City area (Serbia), it was found that the analysed soil had a relatively high proportion of clay and unfavourable physical properties. Additionally, the soil surface horizon is characterized by a medium content of soil organic matter, an acidic pH, a good supply of total nitrogen and available potassium, and a low supply of available phosphorus [47].

Soil fertility and plant nutrition are directly related to the activity of microorganisms, which reflects the level of biological activity and the capacity for nutrient transformation, transport, and metabolism [48]. The changes in the total number of certain systematic and physiological groups of the soil microorganisms and their enzyme activity as induced by different types of minerals, organic, and biological fertilizers, in comparison with the non-fertilized stage, can be used as a parameter in determining optimal fertilization treatment to be applied in plant nutrition [49]. 

The present data on the analysed microbiological parameters in tested soil indicate an increase in total microorganisms, actinomycetes, *Azotobacter* spp., ammonifiers, and dehydrogenase activity in the treatment with integrated use of organic NC fertilizer and biological mixture, compared to the other tested treatments and control. The treatments with separate inorganic NPK and organic NC fertilizers influenced stimulating on the number of fungi, which was also emphasized by other authors [50]. Suliasih and Widawati [51] in a greenhouse study determined that biofertilizer containing azotobacter, aspergillum, and rhizobium, combined with organic fertilizer, produced a higher bacterial population than combined with inorganic fertilizer, adding that the total number of bacteria, including actinomycetes, azotobacter and ammonifiers, was higher in the treatments with no inorganic fertilization in the presence or absence of microbial inoculant. The listed authors emphasized that the tested inoculants had a significant impact on soil total bacteria concentration. Previous authors [52] reported improved microbial population and soil enzymatic activities in the long-term application of biofertilizer (containing N-fixers and P-solubilizers) and organic fertilizer (farmyard manure) in maize crops in relation to the chemical fertilizers. It was also stated that farmyard manure supplies additional organic matter to the soil, thus supporting the survival of rhizosphere microorganisms which conditions the increased soil enzymatic activities, such as the activity of dehydrogenase. On the other hand, research by other authors [53] points out that the combined application of microbial and inorganic fertilizers increases the concentration of bacteria growth without altering their enzymatic activities, maintaining similar nutrient soil conditions, and improving N and P uptake in the growing of lettuce. However, certain previous investigations [54,55,56] have generally shown that both inorganic and organic fertilizers can stimulate the growth of specific microorganism groups by supplying them with nutrients, increasing their total number, and improving their activity. Nevertheless, the authors indicated that inorganic fertilizer alone cannot effect the noticeable increase in the soil microbial abundance. The increased rates of inorganic fertilizers often lead to a reduction in the number of certain groups of microorganisms, except in the number of fungi [57]. The general rule is that fungi dominate in acidic soils [58], especially in conditions of inadequate doses of inorganic fertilizer application. This is partially following the obtained results of the present study, considering that high inorganic doses were not tested, and on the other hand, the tested soil is characterized as acidic. In fertilization practices, some authors [14,59] reported higher sensitivity of soil bacteria in relation to fungi. However, not only inorganic fertilizers could lead to distinct fungal communities in the rhizospheres of crops, but also organic fertilizers. Organic fertilizer input, especially manure, strongly increases fungal abundance but simultaneously decreases fungal diversity, whereby NPK inorganic fertilizer has not so strong effect on fungal diversity, but at the same time has a significant influence on the considerable abundances of fungal phytopathogens [60].

The supporting effect of biofertilizers in inorganic and organic fertilization can be seen from the fact that microorganisms increase the rhizosphere root activity, initiate hormonal activity, and, thus, increase the uptake of plant nutrients [61]. Leafy vegetables, as well as other vegetables such as arugula, require relatively high amounts of nutrition for optimal growth since they have a short cycle. Due to that, the use of different types of fertilizers is common in their cultivation [62]. In the present study, distinct variations were found in values of increases and decreases in the proportions of N, C, P, and K in arugula biomass, in three cutting times (swaths) during one agricultural season of the plant, treated with three different fertilizers and their combination. Moreover, there are not many studies related to the impact of different fertilizer types, such as organic, inorganic, and biological, on the chemical composition of arugula aerial biomass. In general, fertilization treatments that included NPK+MIX, NPK+NC, and NC+MIX enhanced arugula chemical parameters, namely, the content of nitrogen, phosphorus, potassium, and carbon, compared to non-fertilized treatment in all three swaths. In some previous works [23,53,63], the content of mineral components in arugula plants was studied and then the influence of different fertilizers on the chemical composition of arugula seeds, and the influence of different fertilization on the chemical composition of some other leafy vegetables from the Brassicaceae family, such as lettuce. The application of inorganic fertilization and microbial inoculants increases foliar N and P contents and improves their uptake in lettuce, contemporaneously maintaining similar levels of soil nutrients [53]. Similarly, increased contents of P, K, and Mg were reported in lettuce grown with biofertilizer variants as compared to with inorganic fertilizer alone [64]. Morais et al. [65] reported the existence of the arugula plant’s reaction to inorganic fertilization, with potassium, nitrogen, phosphorus and calcium being the first, the second, the third and the fourth nutrient, respectively, most accumulated by the plant shoots at 42 days after transplanting the seedlings. In the research of Barlas et al. [63], the average content of nutrient elements in the arugula plant was found to be 4.32% N, 0.25% P, and 5.13% K. Data on N, P, and K content in the present study for non-fertilized treatment are similar to the mentioned values, whereby the values are higher for the content of N, P, and K in the condition of fertilizing usage. 

In protected plastic tunnel experiments, the significant influence of fertilization treatment was observed on the chemical parameters of arugula seeds and the following: biofertilizers increased the levels of nitrogen in arugula seeds up to 4.93%, whereas, in non-fertilized control plots, it decreased 4.13%; the highest level for phosphorus was 0.15% in arugula seeds was observed when inorganic fertilizers were used, whereby the lowest level of 0.05% was recorded when solely biofertilizers were applied; in treatments with compost and inorganic fertilizers the potassium percentage increased to 1.22%, but in biofertilizer treatment was the lowest—0.88% [23]. 

Experimental results in the present study indicate differences in average values of arugula chemical parameters depending on the fertilization treatments and the time of cutting—swath, during one agricultural season. The values of P and K were almost uniform in the first and the third swaths. Decreased content of P and K was observed in the no-fertilization treatments for the first and the second swaths. Generally, a noticeable impact of all fertilization treatments on tested chemical parameters was observed in the second swath in relation to the first and, especially, the third, except for a slight decrease of nitrogen content in NC+MIX treatment in the second swath compared to the first, though it was higher than in the third. Regarding individual swaths of arugula, the obtained results are in accordance with the previous study [66], where the second cut was significantly more productive compared to the first and third cuts, while the first cut was more productive than the fourth cut. For the first, second, and third cutting times, increased tested chemical parameters in the treatments with inorganic fertilizers may be due to the fact that they contain nutrients in forms that are easily available to plants [23], although there are certain advantages of organic fertilizers which make them a good tool for enhancing plant quality. However, organic matter decomposition and nutrient mineralization occur over a long time, unlike the immediate and rapid nutrient availability from inorganic fertilizer [67]. In addition, it was reported that the nitrogen contained in inorganic fertilizers decomposes at once and could be easily lost, whereas the nitrogen contained in organic fertilizers decomposes slowly and can be more easily retained in the soil [48]. 

The present findings indicate the differences in arugula yield at three cutting times/swaths depending on fertilization applied. In general, the most stimulative effect was manifested in NPK+MIX and NPK+NC treatments, and the most inhibitory—in the treatment without fertilization, in all three swaths, and for total yield. These are in accordance with previous studies [23,64,68]. In the research conducted previously [68], it was concluded that integration of inorganic with biological fertilizers and organic manure enhanced the yield and yield attributes. Likewise, the combined application of the biological inoculants with organic fertilizer helped plants to grow under acidic soil conditions. A significant increase in the green leaves’ dry weight, in four cuttings, when arugula plants were treated with inorganic, organic, and biological fertilizers in relation to non-fertilized treatment, has been reported in previous studies [23]. The increased weight of the plant biomass affected by the treatments with inorganic fertilizers in the second and the first swath may be due to the availability of plant nutrients contained in inorganic fertilizers. It is believed that separate applications of inorganic and organic fertilizers cannot bring a sustainable increase in yields [51]. Accordingly, the application of organic and biological fertilizer, each alone, had no significant effect on the yield and content of nutrients, while the combination of the organic fertilizer and bacterial inoculant could significantly improve them compared with the control. 

## 5. Conclusions

Based on the results obtained in the present study, it was established the different effects of applied inorganic, organic, and biological fertilizers on soil microbiological activity, plant chemical parameters, and yield, in semi-controlled cultivation of arugula. There was an increase in the total microorganisms, actinomycetes, *Azotobacter* spp., and dehydrogenase activity in the variant which included an integrated use of organic NC fertilizer and biological mixture, as well as a noticeable increase in the number of ammonifiers in the mentioned treatment and the treatment with a combination of an inorganic NPK and biological mixture, all compared to the treatment without fertilization and other tested treatments. The number of fungi increased in the treatments with separate inorganic NPK and organic NC fertilization, compared to the non-fertilized and biological mixture variants. In general, a stimulative effect on plant chemical parameters and arugula biomass yield was observed in combined inorganic and biological, organic and biological, and inorganic and organic fertilization treatments, and inhibitory effects in non-fertilized treatment, in all three swaths. A total arugula yield (swath I + swath II + swath III) was much higher in the fertilization treatment with integrated use of biological mixture and inorganic NPK fertilizer (NPK+MIX) in relation to other treatments, which was also observed for noticeable effect of the treatment with organic fertilizer and biological mixture (NC+MIX). The lowest total yield was found in the treatment without fertilization. The total yield in the NPK+MIX treatment was 121%, and in the NC+MIX treatment was 87% higher compared to the non-fertilized variant. Stimulative impact of all fertilization treatments on chemical parameters was observed for the second swath in relation to the first and the third. 

The study presents valuable results obtained in a semi-controlled pot experiment in arugula cultivation under different fertilization systems. According to the conducted research, combined inorganic and biological, organic and biological, and inorganic and organic fertilization treatments could be proposed as optimal fertilization variants in arugula growing. Nevertheless, continuation of this kind of study on larger soil areas is desirable to increase our understanding of the current findings, which will potentially make the production of this vegetable more acceptable from an ecological point of view.

## Figures and Tables

**Table 1:** Fertilization treatments and their abbreviations.

Treatment	Abbreviation
Inorganic Fertilizer (alone)	NPK
Organic Fertilizer (alone)	NC
Inorganic Fertilizer + Organic Fertilizer	NPK+NC
Inorganic Fertilizer + Biological Fertilizer	NPK+MIX
Organic Fertilizer + Biological Fertilizer	NC+MIX
Biological Fertilizer (alone)	MIX
Control	Ø, non-fertilized soil

**Table 2:** Main physical, chemical, and microbiological properties of the soil.

Tested Parameters	
Textural class	Light clay
Granulometric and chemical parameters	Values (mean ± standard deviation)
Silt (%), fractions 0.02–0.002 mm	33.1 ± 0.45
Clay (%), fractions < 0.002 mm	36.6 ± 0.67
Sand (%), fractions > 0.02 mm	30.3 ± 0.26
pH in H_2_O	5.80 ± 0.08
pH in 1M KCl	4.72 ± 0.03
Total N (%)	0.17 ± 0.002
Soil organic matter (SOM, %)	3.30 ± 0.04
Available P (mg 100 g^−1^)	0.20 ± 0.00
Available K (mg 100 g^−1^)	26.70 ± 0.30
Microbiological parameters	
Total number of microorganisms (×10^6^) ^1^	7.04 ± 1.88
Fungi (×10^4^) ^1^	7.09 ± 1.55
Actinomycetes (×10^4^) ^1^	7.56 ± 1.92
*Azotobacter* spp. ^2^	22.67 ± 0.00
Ammonifiers (×10^5^) ^2^	115.50 ± 48.69
DHA (µg TPF g^−1^) ^3^	39.97 ± 4.18

^1^ number of microorganisms per g of absolute dry soil; ^2^ most probable number (MPN) of microorganisms per g of absolute dry soil; ^3^ air-dried soil.

**Table 3:** Effect of the fertilization treatment on tested microbiological parameters.

Fertilization Treatment ^1^	Tested Parameters (Average ± Standard Deviation)					
	Total Number of Microorganisms (×10^6^) ^2^	Fungi (×10^4^) ^2^	Actinomycetes (×10^4^) ^2^	*Azotobacter* spp. ^3^	Ammonifiers (×10^5^) ^3^	DHA (µg TPF g^−1^) ^4^
NPK	6.93 ± 1.27 ^c^	13.15 ± 1.40 ^a^	8.70 ± 0.64 ^b^	30.67 ± 8.08 ^c^	121.67 ± 12.58 ^a^	86.91 ± 1.02 ^e^
NC	10.75 ± 1.46 ^bc^	12.61 ± 1.46 ^a^	12.57 ± 1.71 ^b^	24.00 ± 5.29 ^c^	121.83 ± 3.18 ^a^	88.21 ± 1.53 ^e^
NPK+NC	14.61 ± 1.20 ^ab^	10.79 ± 1.72 ^ab^	12.18 ± 2.11 ^b^	31.00 ± 6.56 ^c^	120.33 ± 10.00 ^a^	104.37 ± 0.51 ^c^
NPK+MIX	14.76 ± 1.08 ^ab^	8.71 ± 1.36 ^ab^	13.82 ± 3.39 ^b^	49.33 ± 9.45 ^b^	133.67 ± 2.75 ^a^	127.11 ± 0.40 ^b^
NC+MIX	17.96 ± 4.78 ^a^	10.15 ± 1.36 ^ab^	19.30 ± 4.27 ^a^	62.00 ± 10.00 ^a^	137.00 ± 2.60 ^a^	134.76 ± 2.15 ^a^
MIX	11.96 ± 0.93 ^b^	7.11 ± 2.04 ^b^	9.26 ± 0.65 ^b^	31.67 ± 5.77 ^c^	123.67 ± 10.61 ^a^	96.71 ± 1.19 ^d^
Ø, non-fertilized	9.53 ± 0.70 ^bc^	7.16 ± 2.16 ^b^	7.92 ± 1.92 ^b^	25.00 ± 0.00 ^c^	118.50 ± 6.06 ^a^	54.17 ± 1.16 ^f^
Statistical analyses	Source of variation					
	Fertilization treatment					
*p* value	***	**	***	***	NSD	***
LSD (0.05)	3.56	3.28	4.20	12.56	13.75	2.25
LSD (0.01)	5.07	4.55	5.83	17.44	19.09	3.13

^1^ fertilization treatments and their abbreviation names are given in Table 1; ^2^ number of microorganisms per g of absolute dry soil; ^3^ most probable number (MPN) of microorganisms per g of absolute dry soil; ^4^ air-dried soil; LSD—least significant differences at *p* = 0.05 and *p* = 0.01; ** and *** indicates statistically significant differences; NSD indicates no significant difference; DMRT at *p* ≤ 0.05—values followed by the same letter in a column are not significantly different.

**Table 4:** Effect of the fertilization treatments on the chemical composition of arugula—swath I.

Fertilization Treatment ^1^	Chemical Composition (%, Dry Biomass) ^2^			
	N	C	P	K
NPK	3.90 ± 0.35 ^a^	33.57 ± 0.29 ^a^	0.44 ± 0.01 ^b^	7.15 ± 0.02 ^c^
NC	5.39 ± 0.99 ^a^	33.74 ± 0.47 ^a^	0.40 ± 0.01 ^bc^	7.08 ± 0.03 ^d^
NPK+NC	5.66 ± 0.58 ^a^	32.65 ± 0.66 ^a^	0.49 ± 0.02 ^a^	7.18 ± 0.01 ^c^
NPK+MIX	5.70 ± 0.18 ^b^	35.17 ± 0.32 ^a^	0.35 ± 0.04 ^cd^	7.80 ± 0.02 ^a^
NC+MIX	5.61 ± 0.33 ^a^	32.93 ± 1.85 ^a^	0.41 ± 0.02 ^b^	7.64 ± 0.01 ^b^
MIX	5.65 ± 0.26 ^a^	34.69 ± 1.10 ^a^	0.37 ± 0.02 ^cd^	6.95 ± 0.04 ^e^
Ø, non-fertilized	5.18 ± 0.13 ^a^	33.93 ± 0.62 ^a^	0.23 ± 0.01 ^f^	6.61 ± 0.01 ^f^
Statistical analyses	Source of variation			
	Fertilization treatment			
*p* value	*	NSD	***	***
LSD (0.05)	1.046	1.960	0.037	0.039
LSD (0.01)	1.452	2.721	0.051	0.054

^1^ fertilization treatments and their abbreviation names are given in Table 1; ^2^ average ± standard deviation; LSD—least significant differences at *p* = 0.05 and *p* = 0.01; * and *** indicate statistically significant differences; NSD indicates no significant difference; DMRT at *p* ≤ 0.05—values followed by the same letter in a column are not significantly different.

**Table 5:** Effect of the fertilization treatments on the chemical composition of arugula—swath II.

Fertilization Treatment ^1^	Chemical Composition (%, Dry Biomass) ^2^			
	N	C	P	K
NPK	6.09 ± 0.08 ^b^	34.53 ± 0.58 ^b^	0.42 ± 0.02 ^c^	7.15 ± 0.02 ^d^
NC	5.70 ± 0.01 ^c^	35.19 ± 0.31 ^ab^	0.42 ± 0.03 ^c^	7.11 ± 0.01 ^e^
NPK+NC	4.21 ± 0.10 ^d^	35.66 ± 0.29 ^ab^	0.50 ± 0.03 ^a^	7.18 ± 0.01 ^c^
NPK+MIX	6.36 ± 0.17 ^ab^	35.89 ± 0.51 ^a^	0.44 ± 0.01 ^bc^	7.87 ± 0.02 ^a^
NC+MIX	6.44 ± 0.15 ^a^	35.33 ± 0.24 ^ab^	0.47 ± 0.02 ^ab^	7.74 ± 0.01 ^b^
MIX	6.10 ± 0.07 ^b^	35.67 ± 0.27 ^ab^	0.38 ± 0.02 ^d^	6.99 ± 0.01 ^f^
Ø, non-fertilized	5.74 ± 0.08 ^c^	36.42 ± 0.32 ^a^	0.21 ± 0.01 ^e^	6.59 ± 0.01 ^g^
Statistical analyses	Source of variation			
	Fertilization treatment			
*p* value	***	**	***	***
LSD (0.05)	0.229	0.814	0.034	0.022
LSD (0.01)	0.319	1.129	0.047	0.031

^1^ fertilization treatments and their abbreviation names are given in Table 1; ^2^ average ± standard deviation; LSD—least significant differences at *p* = 0.05 and *p* = 0.01; ** and *** indicate statistically significant differences; DMRT at *p* ≤ 0.05—values followed by the same letter in a column are not significantly different.

**Table 6:** Effect of the fertilization treatments on the chemical composition of arugula—swath III.

Fertilization Treatment ^1^	Chemical Composition (%, Dry Biomass) ^2^			
	N	C	P	K
NPK	2.66 ± 0.05 ^de^	32.19 ± 0.18 ^b^	0.33 ± 0.02 ^c^	6.81 ± 0.10 ^a^
NC	3.94 ± 0.04 ^b^	33.22 ± 0.23 ^a^	0.35 ± 0.04 ^bc^	6.08 ± 0.04 ^c^
NPK+NC	2.83 ± 0.05 ^d^	32.47 ± 0.26 ^b^	0.38 ± 0.01 ^b^	6.18 ± 0.01 ^c^
NPK+MIX	4.72 ± 0.15 ^a^	33.63 ± 0.37 ^a^	0.33 ± 0.02 ^c^	6.80 ± 0.11 ^a^
NC+MIX	3.40 ± 0.15 ^c^	33.55 ± 0.32 ^a^	0.42 ± 0.01 ^a^	6.66 ± 0.06 ^b^
MIX	3.21 ± 0.06 ^c^	32.20 ± 0.20 ^b^	0.31 ± 0.02 ^c^	6.12 ± 0.03 ^c^
Ø, non-fertilized	2.58 ± 0.03 ^e^	33.62 ± 0.46 ^a^	0.23 ± 0.02 ^d^	6.61 ± 0.02 ^b^
Statistical analyses	Source of variation			
	Fertilization treatment			
*p* value	***	***	***	***
LSD (0.05)	0.191	0.652	0.037	0.113
LSD (0.01)	0.266	0.905	0.051	0.156

^1^ fertilization treatments and their abbreviation names are given in Table 1; ^2^ average ± standard deviation; LSD—least significant differences at *p* = 0.05 and *p* = 0.01; *** indicates statistically significant differences; DMRT at *p* ≤ 0.05—values followed by the same letter in a column are not significantly different.

**Table 7:** Effect of different fertilizers on the yield of arugula aerial biomass.

Fertilization Treatment ^1^	Yield (g pot^−1^, Air Dry Biomass) ^2^			
	Swath I	Swath II	Swath III	Total Biomass
NPK	4.52 ± 0.99 ^b^	5.29 ± 2.55 ^a^	6.47 ± 1.41 ^a^	16.28 ± 4.38 ^b^
NC	4.59 ± 2.06 ^b^	5.99 ± 0.87 ^a^	6.65 ± 1.93 ^a^	17.23 ± 1.74 ^b^
NPK+NC	4.71 ± 0.72 ^b^	5.42 ± 1.62 ^a^	5.10 ± 2.75 ^a^	15.23 ± 1.60 ^b^
NPK+MIX	8.60 ± 0.52 ^a^	7.05 ± 0.55 ^a^	6.68 ± 2.08 ^a^	22.33 ± 3.35 ^a^
NC+MIX	4.83 ± 0.54 ^b^	6.81 ± 1.73 ^a^	7.25 ± 0.54 ^a^	18.89 ± 3.48 ^b^
MIX	4.48 ± 1.76 ^b^	4.31 ± 0.50 ^a^	4.76 ± 0.82 ^a^	13.55 ± 1.88 ^b^
Ø, non-fertilized	3.23 ± 0.97 ^b^	3.59 ± 1.39 ^a^	3.27 ± 1.86 ^a^	10.09 ± 1.43 ^b^
Statistical analyses	Source of variation			
	Fertilization treatment			
*p* value	***	NSD	NSD	*
LSD (0.05)	2.176	3.294	3.969	5.937
LSD (0.01)	3.020	4.496	5.509	8.239

^1^ fertilization treatments and their abbreviation names are given in Table 1; ^2^ average ± standard deviation; LSD—least significant differences at *p* = 0.05 and *p* = 0.01; * and *** indicate statistically significant differences; NSD indicates no significant difference; DMRT at *p* ≤ 0.05—values followed by the same letter in a column are not significantly different.

## Data Availability

The original contributions presented in the study are included in the article, further inquiries can be directed to the corresponding author.
