# Correction: Stanojković-Sebić et al. Response of Arugula to Integrated Use of Biological, Inorganic, and Organic Fertilization. *Microorganisms* 2024, *12*, 1334

**DOI:** 10.3390/microorganisms12091798

**Published:** 2024-08-30

**Authors:** Aleksandra Stanojković-Sebić, Vladimir Miladinović, Olivera Stajković-Srbinović, Radmila Pivić

**Affiliations:** 1 Institute of Soil Science, Teodora Drajzera 7, 11000 Belgrade, Serbia; soils.stanojkovicsebic@gmail.com (A.S.-S.); oliverastajkovic@yahoo.com (O.S.-S.); 2 Institute for Vegetable Crops, Karadjordjeva 71, 11420 Smederevska Palanka, Serbia; vladimir.miladinovic33@gmail.com

In the original publication [1], one co-author, Dr. Olivera Stajković-Srbinović from the Institute of Soil Science, whose contribution to the work was in conceptualization, methodology, and resources, was omitted. In the corrected paper, it is necessary to indicate the named co-author; the correct sequence of author and co-authors of this paper should be as follows:

Aleksandra Stanojković-Sebić ^1^, Vladimir Miladinović ^2^, Olivera Stajković-Srbinović ^1^ and Radmila Pivić ^1,^*

^1^  Institute of Soil Science, Teodora Drajzera 7, 11000 Belgrade, Serbia; soils.stanojkovicsebic@gmail.com (A.S.-S.); oliverastajkovic@yahoo.com (O.S.-S.)
^2^  Institute for Vegetable Crops, Karadjordjeva 71, 11420 Smederevska Palanka, Serbia; vladimir.miladinovic33@gmail.com

*  Correspondence: drradmila@pivic.com

In the author contributions part, the contributions of the newly added co-author should include conceptualization, methodology, and resources, as follows:

Author Contributions: Conceptualization, R.P., A.S.-S. and O.S.-S.; fieldwork, R.P., A.S.-S. and V.M.; methodology, A.S.-S. and O.S.-S.; software, R.P. and A.S.-S.; validation, A.S.-S., R.P. and V.M.; formal analysis, R.P., A.S.-S. and V.M.; investigation, R.P. and A.S.-S.; resources, R.P., A.S.-S., O.S.-S. and V.M.; data curation, A.S.-S. and R.P.; writing—original draft preparation, A.S.-S. and R.P.; writing—review and editing, A.S.-S. and R.P.; supervision, R.P.; project administration, A.S.-S. All authors have read and agreed to the published version of the manuscript.

The authors state that the scientific conclusions are unaffected. This correction was approved by the Academic Editor. The original publication has also been updated.
